# TRPV1 Channel in Human Eosinophils: Functional Expression and Inflammatory Modulation

**DOI:** 10.3390/ijms25031922

**Published:** 2024-02-05

**Authors:** Tobias Weihrauch, Natalie Gray, Daniela Wiebe, Martin Schmelz, Maren M. Limberg, Ulrike Raap

**Affiliations:** 1 Division of Experimental Allergy and Immunodermatology, Faculty of Medicine and Health Sciences, Carl von Ossietzky University Oldenburg, 26129 Oldenburg, Germany; 2 Division of Anatomy, Faculty of Medicine and Health Sciences, Carl von Ossietzky University Oldenburg, 26129 Oldenburg, Germany; 3 Department of Experimental Pain Research, MCTN, Medical Faculty Mannheim, University of Heidelberg, 68167 Mannheim, Germany; 4 Research Center for Neurosensory Science, Carl von Ossietzky University Oldenburg, 26129 Oldenburg, Germany; 5 University Clinic of Dermatology and Allergy, Klinikum Oldenburg, University Oldenburg, 26133 Oldenburg, Germany

**Keywords:** TRPV1, eosinophils, atopic dermatitis, itch

## Abstract

The transient receptor potential vanilloid 1 (TRPV1) is a non-selective cation channel expressed on sensory neurons and immune cells. We hypothesize that TRPV1 plays a role in human eosinophil function and is modulated by inflammatory conditions. TRPV1 expression on human eosinophils was examined by qPCR, flow cytometry, and immunohistochemistry, respectively. TRPV1 functionality was analyzed by investigating calcium flux, apoptosis, modulation by cytokines and acidic pH, and CD69 externalization using flow cytometry. Activation of TRPV1 induced calcium influx and prolonged survival. Although eosinophils were not directly activated by TRPV1 agonists, activation by IL-3 or GM-CSF was mainly restricted to TRPV1-positive eosinophils. TRPV1 surface content was increased by acidic pH, IL-3, IL-31, IL-33, TSLP, TNF-α, BDNF, and NGF-β. Interestingly, TRPV1 was also expressed by eosinophils located in proximity to peripheral nerves in atopic dermatitis (AD) skin. In conclusion, eosinophils express functional TRPV1 channels which are increased by extracellular acidification and AD-related cytokines. Since eosinophils also express TRPV1 in AD skin, our results indicate an important role of TRPV1 for neuroimmune interaction mechanisms in itchy, inflammatory skin diseases, like AD.

## 1. Introduction

Atopic dermatitis (AD) is an inflammatory skin disease which can impair patients’ quality of life at all ages through symptoms such as intense pruritus and chronic eczema. The exact mechanisms of this disorder are still unknown. However, the acute and subacute phases of AD are driven by Th2 cytokines such as interleukin (IL)-13, IL-31, IL-33, and the pro-Th2 cytokine thymic stromal lymphopoietin (TSLP) [1]. The chronic phase is additionally associated with increased serum levels of the Th1 cytokine TNF-α [1]. Leukocytes like eosinophil granulocytes are known to play an essential role in AD [2]. Human eosinophils derive from the myeloid blood cell lineages and constitute about 1–5% of all circulating leukocytes [3]. The first function of eosinophils to be discovered was their role in host defense against helminths and extracellular bacteria [4]. To combat those infections, eosinophils release reactive oxygen species (ROS) and toxic granule proteins such as major basic protein (MBP), eosinophil-derived neurotoxin (EDN), eosinophil cationic protein (ECP), and eosinophil peroxidase [5]. Eosinophils express a variety of cytokine receptors, such as IL-3Rα, GMCSF-Rα, IL31RA/OSMR, IL-33R, TSLPR, TNF-R1 and -R2, TrkA, and TrkB, chemokine receptors such as CCR3, and adhesion molecules on their cell surface [3,6,7,8,9,10]. IL-3Rα and GMCSF-Rα are the receptors for the cytokines IL-3 and granulocyte-macrophage colony-stimulating factor (GM-CSF), which are known to activate eosinophils. This activation is accompanied by the release of granule proteins, such as EDN, which inhibit eosinophil apoptosis [11]. Activation of eosinophils is known to lead to shedding and de novo externalization of the surface protein CD69 [3]. The neurotrophin receptors TrkA (NGF receptor) and TrkB (BDNF receptor) are known to be upregulated in eosinophils of AD patients in comparison to eosinophils of healthy controls [12]. Serum levels of BDNF correlate with disease severity in AD, and it has been reported that eosinophils of AD patients release BDNF in close vicinity to peripheral nerves, which causes outgrowth and branching of nerve fibers [2]. NGF, which is significantly increased in AD [13,14], has been found to be located in the central core of stable granules in eosinophils. The alarmin IL-33, which is the agonist of IL-33R, was also observed to be increased in serum of AD patients. Furthermore, it correlates with disease severity and has been suggested to promote inflammation by being released from damaged skin [15]. TSLP signals through the TSLP receptor complex, composed of TSLPR and IL-7Rα, and is suggested to be an important factor in AD, since serum levels in adults and children are significantly increased [16]. Moreover, polymorphisms of the TSLP gene have been reported to be associated with a higher risk of developing AD [17]. IL-31 is an itch-mediating cytokine which is also released by eosinophils in AD [18]. Since its receptor IL-31RA/OSMR is co-expressed with the TRPV1 ion channel on dorsal root ganglia [19], and Th1 and Th2 cytokines increase TRPV1 expression in human basophils [20] and neurons [1], it is a new candidate for playing an important role in itch. TRPV1 is a non-selective homotetrameric cation channel of the TRP superfamily. Every subunit consists of six transmembrane segments which are joined by intracellular and extracellular loops. The loops connecting the fifth and sixth segment, with a short pore helix in between, form the ion conduction pathway [21,22]. TRPV1 can be activated by noxious stimuli like vanilloids (capsaicin), high temperature, extracellular acidification [21,23,24], chemokines [25], or inflammatory molecules such as histamine [26], adenosine triphosphate (ATP) [27], Bradykinin [28], Prostaglandin [29], and nerve growth factors like NGF [30]. The activation of TRPV1 leads to depolarization and calcium influx which then regulates cell functions like nociception, production and release of cytokines, phagocytosis, and cell migration [20,31,32,33]. This, in turn, influences inflammation, pain, and pruritus [26,34,35]. TRPV1 is known to be expressed on sensory neurons [21]. However, TRPV1 has also been identified in different non-neuronal cells like keratinocytes, epithelial cells [36,37,38,39], and immune cells such as human mast cells [40], dendritic cells [41], T cells [42], basophils [20], neutrophils [43,44], monocytes, and macrophages [45]. Although the expression of TRPV1 on eosinophils was demonstrated by Zhu et al. [46], the functional role of the channel in eosinophils is not known.

The aim of this study was to investigate the expression, modulation, and functionality of TRPV1 on human eosinophils to gain new insights into its possible contribution to itchy skin diseases such as AD.

## 2. Results

### 2.1. TRPV1 Is Expressed on RNA and Protein Levels in Human Eosinophils

**Figure 1:**
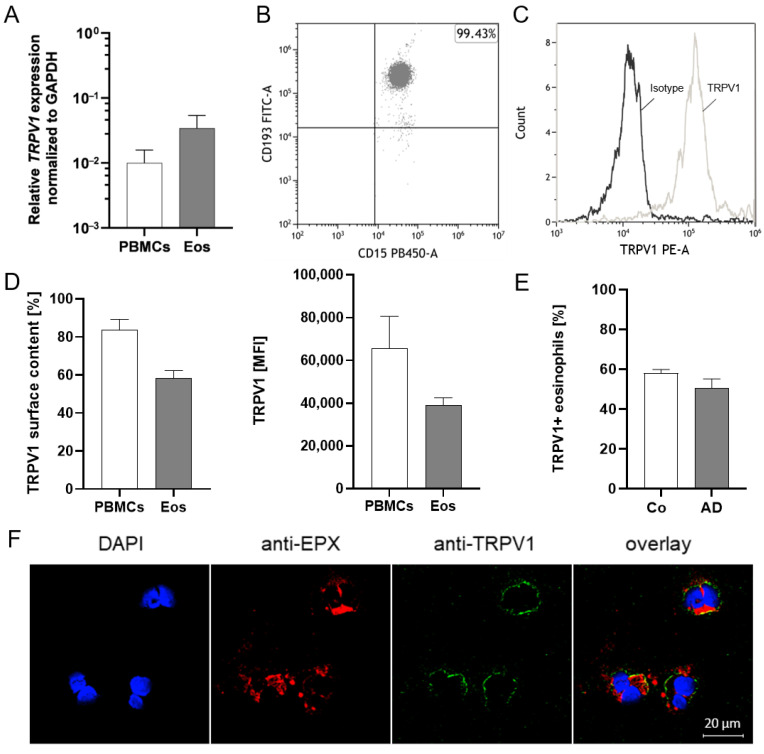
TRPV1 mRNA and protein expression on human peripheral blood eosinophils. (**A**) Total RNA was purified from human peripheral blood eosinophils and PBMCs from healthy individuals and reverse transcribed. TRPV1 mRNA expression levels were determined through quantitative real-time PCR. Measurements were performed in duplicate. Data were normalized to the expression levels of the housekeeping gene GAPDH (eosinophils n = 3; PBMCs n = 3; ±SEM). (**B**) For the protein expression analysis, eosinophil purity was determined by the percentage of CD193+ and CD15+ cells of CD16- cells (one representative scatter dot plot of three is shown). Eosinophils from healthy subjects were stained with the TRPV1-PE antibody. The gate for TRPV1+ cells was set according to the FMO control (not shown). (**C**) The isotype control confirmed that the TRPV1-PE antibody does not bind unspecifically to eosinophils (one representative histogram of three). (**D**) Percentage and mean fluorescence intensity (MFI) of TRPV1+ eosinophils and PBMCs from healthy subjects assessed by flow cytometry (Eos n = 6 and PBMCs n = 3; ±SEM). (**E**) Percentage of TRPV1+ eosinophils from peripheral blood of healthy controls (Co) and AD patients (healthy controls n = 6; AD n = 5; ±SEM). (**F**) Microscopic section of TRPV1+ human peripheral blood eosinophils from healthy individuals (n = 3). Eosinophils are shown at 40× magnification (see scale bar at the bottom right). Nuclei were stained with DAPI (blue), the eosinophil marker eosinophil peroxidase (EPX) with anti-EPX (red), and the TRPV1 channel with anti-TRPV1 (green). The right panels display an overlay of all fluorescence channels. Colocalizations appear yellow.

TRPV1 mRNA expression in human peripheral blood eosinophils and PBMCs (positive control) was examined through qPCR. Our data show that TRPV1 mRNA expression in eosinophils is higher than in PBMCs in all donors (Figure 1A). To further confirm the expression on the protein level, TRPV1 surface expression of highly purified human eosinophils (CD15+, CD193+, and CD16- cells) was analyzed through flow cytometry (Figure 1B and Figure S1). The specificity of the TRPV1-PE antibody was confirmed through use of an isotype control (Figure 1C). The results revealed that 59% of eosinophils expressed TRPV1 at the cell surface, with a mean fluorescence intensity of about 40,000. The surface expression on eosinophils is lower than on PBMCs (Figure 1D). Further, we compared TRPV1 surface expression on peripheral blood eosinophils from healthy controls and AD patients and could not find significant differences (*p* = 0.1289) (Figure 1E). Moreover, we observed that the TRPV1 channel (anti-VR1, green) is found on a subset of purified human peripheral blood eosinophils (anti-EPX, red) (Figure 1F).

### 2.2. Activation of TRPV1 Induces Calcium Influx

**Figure 2:**
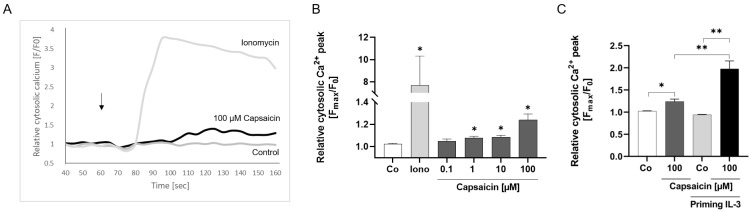
Calcium flux after activation of TRPV1 in eosinophils. (**A**) Purified eosinophils from healthy subjects were labeled with Fluo-4 FITC-A. Capsaicin, ionomycin, or RPMI medium were applied 60 s after starting the measurement (arrow). The median of fluorescence intensity at two time points (at 40 s before application and peak of fluorescence intensity after application) was used for calculating the changes in intracellular calcium levels. A representative graph of the average changes in intracellular calcium over time is shown for ionomycin, 100 µM capsaicin, and the negative control. (**B**) Relative cytosolic calcium concentration peak in eosinophils after the application of RPMI medium (neg. control; Co), ionomycin (Iono), and capsaicin (0.1, 1, 10, 100 µM) (n = 4). (**C**) Relative cytosolic calcium concentration peak in eosinophils after the application of RPMI medium (neg. control; Co) or 100 µM capsaicin with (n = 4) and without (n = 4) eosinophils primed with IL-3 (10 ng/mL) for 20 min at 37 °C and 5% CO_2_ (* = *p* < 0.05; ** = *p* < 0.01; ±SEM).

Since TRPV1 activation contributes to calcium flux in human basophils [20], we analyzed the functionality of TRPV1 on eosinophils by measuring transient changes of intracellular calcium levels after channel activation via different concentrations of the specific agonist capsaicin (Figure 2A,B). We used the calcium ionophore ionomycin as a positive control which induces maximum calcium transients (Figure 2A,B). Eosinophils were labeled with Fluo-4, which intensifies the fluorescence of cells by binding to intracellular calcium ions. After measurement through flow cytometry, the median of fluorescence intensity was calculated before (at baseline before stimulation) and at the peak of fluorescence intensity after application of capsaicin. Our data show that capsaicin induced a significant calcium influx (Figure 2A,B) at 1 µM (*p* = 0.0104), 10 µM (*p* = 0.0124), and 100 µM (*p* = 0.0315) but not at 0.1 µM of capsaicin (Figure 2B). These responses were delayed by some seconds as compared to application of ionomycin, which induced an instantaneous calcium influx (*p* = 0.0148). Furthermore, we observed that eosinophil priming with IL-3 also led to calcium influx with 100 µM capsaicin (*p* = 0.0012), which even exceeded the capsaicin response in untreated eosinophils (*p* = 0.0080) (Figure 2C).

### 2.3. TRPV1 Activation Has an Antiapoptotic Effect on Human Eosinophils

**Figure 3:**
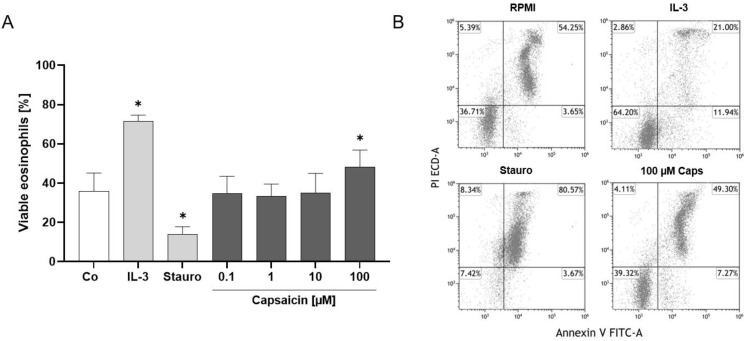
Detection of apoptosis after TRPV1 activation. (**A**) Percentage of viable eosinophils from healthy subjects after incubation with capsaicin (0.1, 1, 10, 100 µM), IL-3 (10 ng/mL) as an antiapoptotic, staurosporine (1 µM) as a proapoptotic, or RPMI medium as the negative control (Co) for 24 h at 37 °C and 5% CO_2_. Eosinophils were stained with Annexin V and propidium iodide, and fluorescence was measured by flow cytometry (n = 5; * = *p* < 0.05; ±SEM). (**B**) Representative dot plots of the negative control, anti- and proapoptotic control, and 100 µM capsaicin. Viable eosinophils are shown in the lower left, apoptotic eosinophils in the lower right, late apoptotic eosinophils in the upper right, and necrotic eosinophils in the upper left quadrant.

Previous studies demonstrated that TRPV1 can exhibit both pro- and antiapoptotic effects [20,47,48]. Therefore, we investigated the impact of TRPV1 on human eosinophil survival and death by performing an annexin V and propidium iodide staining after 4 h and 24 h of stimulation with different capsaicin doses, IL-3 as an antiapoptotic, and staurosporine as the proapoptotic control. Interestingly, we observed that TRPV1 has an antiapoptotic effect after 24 h, as more viable eosinophils were present after activation with 100 µM capsaicin than in the RPMI medium negative control (*p* = 0.0452) (Figure 3A,B). The IL-3 (*p* = 0.0113) and staurosporine (*p* = 0.0283) controls worked as expected, with significantly higher or lower numbers of viable eosinophils being present than in the negative control (Figure 3A,B). We did not observe a significant change in eosinophil viability after stimulation with capsaicin and IL-3 after 4 h (Figure S2).

### 2.4. Eosinophil Surface Expression of TRPV1 Is Linked to Activation Status

**Figure 4:**
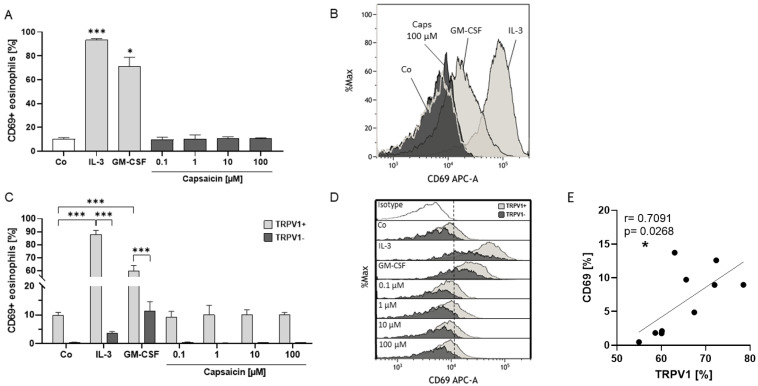
Externalization of the activation marker CD69 after TRPV1 activation. (**A**) Percentage of CD69+ eosinophils after stimulation with the TRPV1 agonist capsaicin (0.1, 1, 10, 100 µM), IL-3 (10 ng/mL), GM-CSF (10 ng/mL) as positive controls, and RPMI medium as the negative control (Co) for 24 h at 37 °C and 5% CO_2_ (n = 3; * = *p* < 0.05, *** = *p* < 0.001; ±SEM). TRPV1 expression was assessed through flow cytometry. (**B**) Representative histogram of CD69 externalization on human peripheral blood eosinophils from healthy individuals after stimulation with IL-3, GM-CSF, capsaicin (Caps; 100µM), and RPMI medium as a negative control (Co). (**C**) Percentage of CD69+ TRPV1+ and CD69+ TRPV1- eosinophils after stimulation (n = 3; *** = *p* < 0.001; ±SEM). (**D**) Representative histograms of CD69 expression on TRPV1+ (bright) and TRPV1- (dark) eosinophils after stimulation. Purified eosinophils were stained with CD69-APC and TRPV1-PE antibodies. (**E**) Correlation of relative TRPV1 and CD69 surface expression on unstimulated eosinophils from healthy individuals (n = 10; * = *p* < 0.05).

Subsequently, we assessed the activation status of human eosinophils by analyzing CD69 surface externalization through flow cytometry. The activation of TRPV1 by capsaicin had no impact on CD69 surface content, while the IL-3 and GM-CSF positive controls induced significantly higher CD69 externalization (Figure 4A,B). Interestingly, TRPV1+ eosinophils exhibited higher CD69 amounts on the surface (Figure 4C,D) than TRPV1- eosinophils after stimulation with IL-3 (*p* < 0.001) and GM-CSF (*p* < 0.001). Moreover, we observed that only TRPV1+ eosinophils exhibited significantly higher CD69 surface content after stimulation with IL-3 (*p* < 0.001) and GM-CSF (*p* < 0.001) compared to the negative control. In contrast, even after IL-3 or GM-CSF treatment, the level of CD69 surface expression in TRPV1- eosinophils did not exceed the baseline expression in TRPV1+ eosinophils. Furthermore, we found a correlation between the percentage of TRPV1+ and CD69+ unstimulated eosinophils (r = 0.0791; *p* = 0.0268) (Figure 4E).

### 2.5. TRPV1 Surface Expression Is Modulated by AD-Related Cytokines and pH

**Figure 5:**
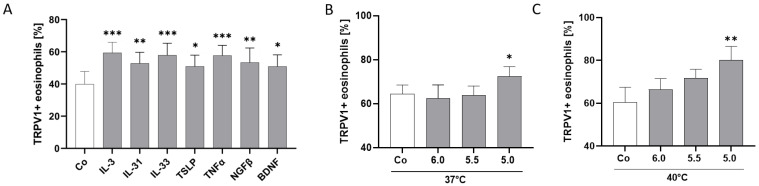
TRPV1 is upregulated by AD-related cytokines, neurotrophins, and extracellular acidification. (**A**) Percentage of TRPV1+ eosinophils from healthy individuals assessed through flow cytometry after stimulation with IL-3 (10 ng/mL), IL-31 (10 ng/mL), IL-33 (10 ng/mL), TSLP (10 ng/mL), TNF-α (10 ng/mL), NGF-β (10 ng/mL), BDNF (50 ng/mL), or RPMI medium (negative control; Co) for 4 h at 37 °C and 5% CO_2_ (n = 5; * = *p* < 0.05, ** = *p* < 0.01, *** = *p* < 0.001; ±SEM). (**B**) Percentage of TRPV1+ eosinophils after incubation for 4 h at 37 °C and pH 5.0, 5.5, 6.0, or 7.0 (Co) (n = 3). (**C**) Percentage of TRPV1+ eosinophils after incubation for 4 h at 40 °C and pH 5.0, 5.5, 6.0, or 7.0 (Co) (n = 3; * = *p* < 0.05, ** = *p* < 0.01; ±SEM).

Since TRPV1 has been found to be sensitized by different mediators [20,48,49,50,51], we wanted to investigate if cytokines and neurotrophins associated with AD and inflammation can modulate TRPV1 surface expression on human eosinophils. Additionally, we investigated TRPV1 modulation through extracellular acidification and capsaicin. Remarkably, our data clearly demonstrated that IL-3 (*p* = 0.0007), IL-31 (*p* = 0.0022), IL-33 (*p* < 0.001), TSLP (*p* = 0.0105), TNF-α (*p* < 0.001), NGF-β (*p* = 0.0014), and BDNF (*p* = 0.0111) induced a higher expression of TRPV1 on the surface than in the unstimulated eosinophils (Figure 5A). There was no significant change in expression levels after stimulation with IL-13 (Figure S3A). TRPV1 surface expression was not only upregulated by cytokines but also by extracellular acidification. A pH of 5.0 increased TRPV1 surface content significantly after 4h of incubation at 37 °C (*p* = 0.0456) (Figure 5B) and 40 °C (*p* = 0.0044) (Figure 5C). Even if not significant, the effect of pH 5.0 seems to be even higher at 40 °C than at 37 °C (Figure 5B,C). However, activation of TRPV1 through different doses of capsaicin did not change the TRPV1 surface content of human eosinophils (Figure S3B).

### 2.6. TRPV1 Expression on Eosinophils in AD Skin

**Figure 6:**
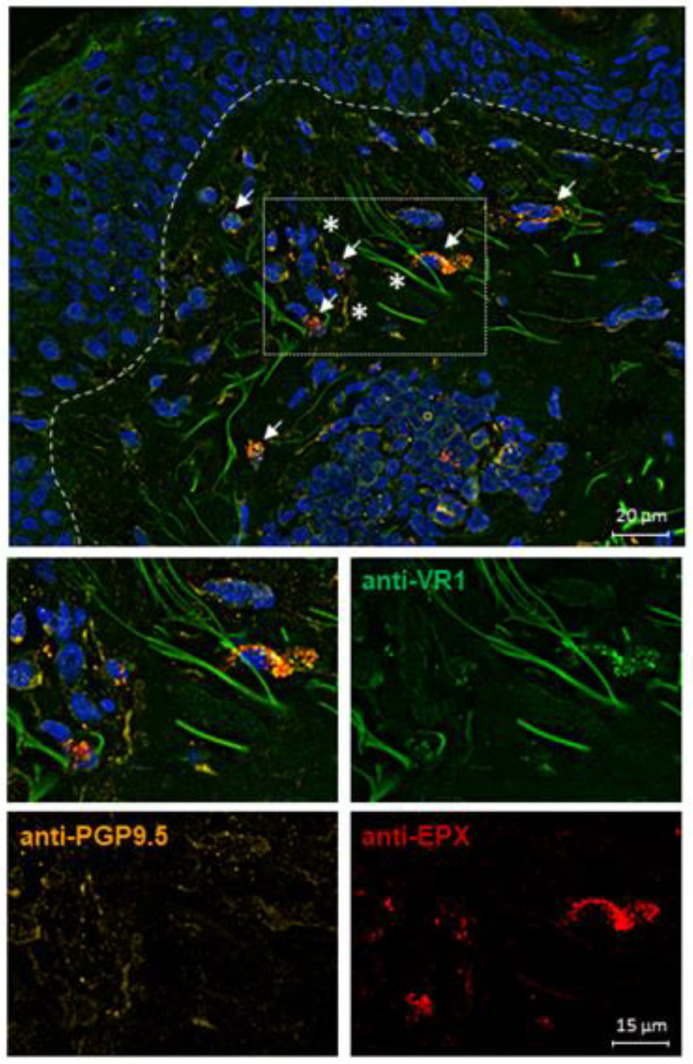
Human eosinophils express TRPV1 in AD skin. Skin sections obtained from AD patients were fixed with methanol and stained with anti-TRPV1 (green), anti-EPX (red) as the eosinophil marker, and anti-PGP9.5 (orange) as the neuronal marker. Cell nuclei were labeled with DAPI (blue). Arrows point to TRPV1+ eosinophils and stars label nerve fibers. TRPV1 expression was analyzed at 40× magnification through fluorescence microscopy. Representative staining out of n = 3 AD patients.

Since we showed that AD-related cytokines upregulate TRPV1 surface expression on human peripheral blood eosinophils, we investigated TRPV1 expression in symptomatic skin of atopic dermatitis patients (n = 3) by staining skin sections. Interestingly, virtually all eosinophils in AD skin expressed TRPV1 and were additionally found to be located in close proximity to peripheral nerves (Figure 6). In contrast, we did not find any eosinophils in skin samples of healthy individuals, as expected (Figure S4).

## 3. Discussion

Our study extends the knowledge on TRPV1 on human peripheral blood eosinophils by verifying the functional role of its expression. TRPV1 expression in eosinophils was increased by inflammatory mediators such as IL-3, IL-31, IL-33, BDNF, NGF-β, TNF-α, and TSLP. Even though eosinophils were not directly activated by TRPV1 agonists, their activation by IL-3 or GM-CSF was mainly restricted to TRPV1+ eosinophils, suggesting that TRPV1 expression is linked to their priming. We hypothesize that such priming may not only contribute to inflammation but, in combination with the close spatial proximity to skin nerves, also to itch.

In accordance with Zhu et al., where protein expression of TRPV1 was detected through Western blot analysis, we confirmed these findings by flow cytometry. Flow cytometry allows for accurate quantification of TRPV1+ cells and precisely detects the density of TRPV1 on cells through calculation of the MFI. It also allows for multiparameter analysis and the identification of subpopulations. In this regard, we could further show that TRPV1+ eosinophils express the activation marker CD69 in higher amounts than TRPV1- eosinophils and that TRPV1 surface expression even correlates with CD69 externalization. Moreover, activation by IL-3 and GM-CSF was virtually restricted to TRPV1+ eosinophils. TRPV1 might indirectly play a role in cell activation by involving other receptors. This has also been shown in CD4+ T cells where stimulation of CD3 and CD28 did not lead to cell activation when TRPV1 was inhibited [42]. Although TRPV1 does not directly activate eosinophils, it appears to facilitate activation and upregulation of TRPV1 expression by cell-activating cytokines. We found that IL-3 may further enhance these responses, as the cytokine was observed to increase TRPV1 surface expression and intensify calcium influx upon channel activation. In basophils, IL-3 increases the content of TRPV1 on the surface by altering channel distribution [20].

Functionally, we were able to show that activation of TRPV1 channels induces calcium influx in human peripheral blood eosinophils and prolongs their survival. Activation of TRPV1 through 100 µM of capsaicin induced a significant change in intracellular calcium levels. These results differ from the findings of Zhu et al. [46], where calcium influx was not detected in eosinophils. However, Zhu et al. conducted the calcium flux experiments in a cuvette with 10^6^ Fura-2-labeled eosinophils after stimulation with 100 µM capsaicin [46], whereas we performed the calcium influx experiments with varying doses of capsaicin using flow cytometry. Thus, the diverging results might be due to the different methods, as the results of single cell measurement are being compared to data acquired from an entire cell suspension. Thus far, rises of intracellular calcium in immune cells through TRPV1 activation have been described for basophils [20], macrophages, dendritic cells, and T cells but not for neutrophils [41,42,52]. Moreover, we were able to demonstrate that activation of TRPV1 with 100 µM of capsaicin has an antiapoptotic effect after 24 h of stimulation. Previously, proapoptotic effects on both basophils [20] and TRPV1-transfected HeLa cells [47] and antiapoptotic, proliferative effects in epithelial cells have been described [48]. Yang et al. reported that activation of TRPV1 by capsaicin leads to transactivation of EGFR and subsequent MAP kinase pathway-induced proliferation [48]. A similar mechanism in eosinophils is conceivable, even if the downstream signals have not yet been analyzed.

We also demonstrated that cytokines, which play an important role in skin inflammation, and an acidic pH can upregulate TRPV1 at the plasma membrane of eosinophils. Surface expression significantly increased after stimulation with IL-3, IL-31, IL-33, BDNF, NGF-β, TNF-α, and TSLP. IL-31 is already known to upregulate TRPV1 expression in DRG [49], and serum levels correlate with disease severity in AD [53,54]. IL-33 was found to be overexpressed in keratinocytes of AD patients [55] and increases the TRPV1 surface content on basophils [20]. IL-33 potentiates TRPV1 on neurons indirectly by inducing the release of IL-31 from Th2 lymphocytes, mast cells, and eosinophils [1]. IL-13 increases TRPV1 in bronchial epithelia and lungs in mice [50], but we could not observe upregulation on eosinophils after IL-13 stimulation (Figure S1A). The pro-Th2 cytokine TSLP is known to promote the production of Th2 cells, which then release even greater amounts of IL-13 [56]. However, according to our data, TRPV1 upregulation through TSLP probably occurs directly, since TSLP but not IL-13 potentiates TRPV1 expression in eosinophils. The Th1 cytokine TNF-α which, in turn, triggers secretion of TSLP in human keratinocytes [57] also potentiates TRPV1 expression in eosinophils. The upregulation of TRPV1 through TNF-α has previously been described for basophils [20] and rat DRG [51]. This upregulation further facilitates the simultaneous insertion of TRPV1 and TRPA1 into the plasma membrane of rat neurons [58]. Furthermore, we discovered that TRPV1 is also potentiated by the neurotrophins BDNF and NGF-β. In HEK293 cells, increased membrane insertion of TRPV1 after NGF stimulation occurs due to TrkA, PI3 kinase, and Src kinase activation [30]. Recently, we also observed an upregulation of TRPV1 surface content through NGF-β on basophils [20]. The impact of BDNF on TRPV1 expression has been investigated on tracheal-specific TrkB+ neurons in a mouse model [59]. Transient exposure of the trachea to BDNF caused a significant increase in TRPV1 mRNA expression in these neurons for up to one week. Continuous administration of BDNF further resulted in a stronger calcium influx in response to the TRPV1-specific agonist capsaicin than in neurons of control mice, suggesting an upregulated protein expression of TRPV1 through BDNF [59]. Correlation of serum levels with disease severity in AD has been reported for both the neurotrophins, BDNF and NGF [2,60,61]. BDNF is released by eosinophils in close vicinity to peripheral nerves, which then causes an outgrowth of nerve fibers [2]. However, not only cytokines but also a low pH of 5.0 can upregulate TRPV1 on the cell surface of eosinophils, which seems to be further enhanced by higher temperatures. It has already been reported that extracellular acidification activates and upregulates TRPV1 in human esophageal epithelial cells and rat DRG neurons [62,63]. We recently showed that extracellular acidification also potentiates TRPV1 surface content in basophils [20]. Since we know that inflammation induces an acidic environment through production of lactate [64], upregulation of TRPV1 on eosinophils in local skin inflammation is conceivable. These data show that TRPV1 can be modulated by a variety of factors. It has also been reported that UV-C irradiation can alter TRPV1 expression on the RNA level by downregulating the channel in human skin [65].

We further performed immunofluorescence staining showing that TRPV1 is expressed on eosinophils in the skin of AD patients located in close proximity to sensory nerve fibers. Since we did not find any differences in TRPV1 surface expression of peripheral blood eosinophils from healthy subjects and AD patients and because eosinophils are only present in lesional AD skin, we hypothesize that TRPV1 might be upregulated through inflammatory mediators locally released by nerves and other immune cells. We previously demonstrated that eosinophils interact with neurons by inducing an outgrowth and branching of nerve fibers via BDNF [2]. In this regard, further investigations of cytokine release and eosinophil chemotaxis after TRPV1 activation might bring new insights into the role of TRPV1 in causing itch or even pain, as an association between TRPV1 and pain has been shown, for instance, in endometriosis [66].

In summary, human peripheral blood eosinophils express functional TRPV1 channels, leading to calcium influx and prolonged survival after channel activation. Furthermore, activation by IL-3 and GM-CSF was grossly restricted to TRPV1 positive eosinophils and extracellular acidification. Further, cytokines such as IL-3, IL-31, IL-33, TSLP, TNF-α, NGF-β, and BDNF increased eosinophil TRPV1 expression. We confirmed that TRPV1 expression occurs in eosinophils in close proximity to peripheral nerves in the skin of AD patients. Future investigations will focus on the possible synergy of TRPV1 with IL-31RA/OSMR, the colocalized expression of which have been described on peripheral nerves. This will help to gain better insights into a possible positive feedback loop mechanism between eosinophils and sensory nerves for pruritus and skin inflammation.

## 4. Materials and Methods

### 4.1. Patient Materials

All samples were collected after informed consent was obtained. Peripheral venous blood was collected from healthy controls with no personal history of allergies or other atopic diseases and from AD patients without immunosuppressive treatment. Skin samples were collected from healthy individuals and from AD patients in chronic stages of the disease without immunosuppressive treatment within the last 2 weeks. Patient materials were collected at the Department of Dermatology and Allergy of Human Medicine Clinic Oldenburg (approved by the local medical ethics committee, University of Oldenburg, ref.# 2017-106, ref.# 2017-109, ref.# 2021-025, and ref.# 2021-078). Peripheral blood mononuclear cells (PBMCs) from healthy blood donors (DRK Blutspendedienst, Springe, Germany; Department of Dermatology and Allergy of Human Medicine Clinic Oldenburg) were isolated by density gradient centrifugation and used as a control for TRPV1 mRNA and protein expression analysis.

### 4.2. Isolation of Human Peripheral Blood Eosinophils

Eosinophils were purified from EDTA blood by immunomagnetic negative selection (EasySep™ Direct Human Eosinophil Isolation Kit, Stem Cell Technologies, Grenoble, France) using half the amounts of RapidSpheres and Isolation Cocktail recommended in the manufacturer’s protocol. The viability was determined by flow cytometry analysis using 7-AAD (Miltenyi Biotec, Bergisch Gladbach, Germany) and was found to be ≥99%. Human eosinophils were identified using CD15-PB, CD16-APC-A750 (Beckman Coulter, Brea, CA, USA), and CD193-FITC (Miltenyi Biotec, Bergisch Gladbach, Germany) antibodies. Isolated eosinophils had a median purity of 95.8%. For cytospins, cells were washed with PBS (Carl Roth, Karlsruhe, Germany) and centrifuged onto object slides using the Cytospin 4 Centrifuge (Thermo Scientific, Darmstadt, Germany).

### 4.3. Flow Cytometry Analysis of TRPV1 and CD69 Externalization

To assess CD69 externalization after TRPV1 activation, purified eosinophils were stimulated with 0.1, 1, 10, or 100 µM capsaicin (Merck, Darmstadt, Germany), with IL-3 (10 ng/mL) (PeproTech, Cranbury, NJ, USA) and GM-CSF (10 ng/mL) (BioLegend, Amsterdam, the Netherlands) as positive controls, for 24 h at 37 °C and 5% CO_2_ in RPMI medium (containing 10% FCS and 1% PenStrep) (VWR International, Leuven, Belgium). To analyze CD69 and TRPV1 expression, eosinophils were stained with CD69-APC (Miltenyi Biotec, Bergisch Gladbach, Germany) and TRPV1-PE (Biozol, Eching, Germany) antibodies. FMO controls were stained without the CD69-APC or TRPV1-PE antibody, respectively. To exclude unspecific binding, cells were also stained with a PE-Rabbit Isotype Control (ab37407, Abcam, Cambridge, UK) or REA APC isotype control (Miltenyi Biotec, Bergisch Gladbach, Germany). Measurement was performed after 10 min of incubation in the dark on the CytoFlexS platform (Beckman Coulter, Brea, CA, USA). CD193-FITC, CD69-APC, CD15-PB, and CD16-APC-A750 were compensated using the MACS Comp Bead Kit anti-REA (Miltenyi Biotec, Bergisch Gladbach, Germany) for the REA antibodies and the MACS Comp Bead Kit anti-mouse Igκ (Miltenyi Biotec, Bergisch Gladbach, Germany) for the remaining antibodies, according to the manufacturer’s protocol.

### 4.4. RNA Isolation and qPCR

Total RNA was isolated from highly purified human eosinophil granulocytes (10^6^ cells) and PBMCs using the High Pure RNA Isolation Kit (Roche, Mannheim, Germany), according to the manufacturer’s protocol. RNA integrity was further assessed through analysis using the Tape Station (Tape Station 4150, Agilent Technologies, Waldbronn, Germany). The Transcriptor First Strand cDNA Synthesis Kit (Roche, Mannheim, Germany) was used for cDNA synthesis. TRPV1 mRNA expression in eosinophils and PBMCs was determined through qPCR using the FastStart Essential SYBR Green Master Mix (Roche, Mannheim, Germany). The housekeeping gene GAPDH was used as a reference gene for relative quantification. Primers for human TRPV1 (NM_080704.3) (ThermoFisher Scientific, Waltham, MA, USA) were as follows: forward, 5′-AGAGTCACGCTGGCAACC-3′; reverse, 5′-GGCAGAGACTCTCCATCACAC-3′. Primers for human GAPDH (NM_002046.5) (ThermoFisher Scientific, Waltham, MA, USA) were as follows: forward, 5’-AGCCACATCGCTCAGACAC-3′; reverse, 5′-GCCCAATACGACCAAATCC-3′. Gene-specific PCR products were measured with the LightCycler 96 (Roche, Mannheim, Germany) for 45 cycles using the following parameters: pre-incubation, 600 s at 95 °C; denaturation, 10 s at 95 °C; annealing, 10 s at 60 °C; and elongation, 10 s at 72 °C. Melting curve analysis was performed to exclude nonspecific amplification. The quantification cycle (Cq) values of *TRPV1* and *GAPDH* were used for calculating the relative quantity (RQ) via the ΔCq method [RQ = 2^−(ΔCq)^].

### 4.5. Immunofluorescence Staining

Eosinophil cytospins (1 × 10^5^ cells) and skin sections from AD patients and healthy controls were stained according to the protocol we recently described [20]. We used an EPX Alexa Fluor 647 antibody (BioTechne, Wiesbaden, Germany) to detect eosinophils (1:200 dilution in blocking solution).

### 4.6. Calcium Flux Experiments

Experiments to analyze changes in calcium flux were performed according to the same procedure as described recently [67]. Purified eosinophils were labeled with 3 µM Fluo-4 (Molecular Probes, Eugine, OR, USA) and stimulated with 0.1, 1, 10, or 100 µM capsaicin (Merck, Darmstadt, Germany) during the measurement. Ionomycin (500 nM) (ThermoFisher Scientific, Waltham, MA, USA) was used as the positive control and RPMI medium (VWR International, Leuven, Belgium) as the negative control. For statistical analysis, the factor of the intracellular fluorescence was calculated through comparing the intensity peak after application of stimulants at 60 s to the baseline (mean value from 40 to 45 s). This experiment was also conducted with 100 µM capsaicin after eosinophil priming with IL-3 (10 ng/mL) (PeproTech, Cranbury, NJ, USA) for 20 min at 37 °C and 5% CO_2_.

### 4.7. Apoptosis Assay

Purified eosinophils were stained with annexin V and propidium iodide (Apoptosis Detection Kit, Beckman Coulter, Brea, CA, USA) to assess apoptosis after 4 and 24 h of incubation. Staurosporine (1 µM) (ThermoFisher Scientific, Waltham, MA, USA) served as the proapoptotic, IL-3 (10 ng/mL) (PeproTech, Cranbury, NJ, USA) as the anti-apoptotic, and RPMI medium (VWR International, Leuven, Belgium) as the negative control. Apoptotic stages were determined through flow cytometry.

### 4.8. Stimulation of Eosinophils with Cytokines and Incubation at Varying pH and Temperatures

Purified eosinophils were resuspended in RPMI medium (containing 10% and 1% PenStrep) (VWR International, Leuven, Belgium). Eosinophils were stimulated for 4 h at 37 °C and 5% CO_2_ with IL-3 (10 ng/mL), IL-13 (50 ng/mL), IL-33 (10 ng/mL), TSLP (10 ng/mL), NGF-β (10 ng/mL), BDNF (50 ng/mL), TNF-α (10 ng/mL), or IL-31 (10 ng/mL) (PeproTech, Cranbury, NJ, USA). Eosinophils were also incubated without any stimulants for 4 h at 37 °C and 40 °C and at pH 5.0, 5.5, 6.0, or 7.0. The pH value of the medium was adjusted with HCl (Sigma-Aldrich, St. Louis, MO, USA) and NaOH (Carl Roth, Karlsruhe, Germany) and determined with a pH electrode.

### 4.9. Statistical Analysis

The qPCR data from the LightCycler96 (Roche, Mannheim, Germany) were analyzed with the LightCycler96 SW 1.1 software. All data from the CytoFlexS platform were analyzed using the Kaluza software version 2.1.1 (Beckman Coulter, Brea, CA, USA). GraphPad Prism 8.0.3 (GraphPad Software, San Diego, CA, USA) was used for statistical analyses. All values are presented as mean ± SEM. Normal Gaussian distribution of data was examined by performing a Shapiro–Wilk normality test. Normally distributed data were analyzed by a parametric paired or unpaired two-tailed *t*-test or one-way ANOVA, and *p* values were considered to be statistically significant if they were < 0.05 (* *p* < 0.05, ** *p* < 0.01, *** *p* < 0.001). The association between TRPV1 and CD69 surface content was performed using Pearson correlation analysis. 

## Data Availability

Data are contained within the article and supplementary materials.

## Supplementary Materials

The supporting information can be downloaded at: https://www.mdpi.com/article/10.3390/ijms25031922/s1.

## Abbreviations

AD	Atopic dermatitis
ATP	Adenosine triphosphate
BDNF	Brain-derived neurotrophic factor
Ca	Calcium
CCR3	CC chemokine receptor type 3 (CD193)
cDNA	Complementary deoxyribonucleic acid
CD16	Cluster of differentiation 16; FcγRIII
CD69	Cluster of differentiation 69
DAPI	4’,6-diamidino-2-phenylindol
ECP	Eosinophil cationic protein
EDN	Eosinophil-derived neurotoxin
EDTA	Ethylenediaminetetraacetic acid
EGFR	Epidermal growth factor receptor
EPX	Eosinophil peroxidase
ER	Endoplasmic reticulum
FCS	Fetal calf serum
Fig	Figure
FMO	Fluorescence minus one
GAPDH	Glyceraldehyde-3-phosphate dehydrogenase
GM-CSF	Granulocyte/macrophage-colony-stimulating factor
h	Hours
IL	Interleukin
Iono	Ionomycin
MAP	Mitogen-activated protein
MBP	Major basic protein
MFI	Mean fluorescence intensity
NGF	Nerve growth factor
OSMR	Oncostatin M receptor
PBMC	Peripheral blood mononuclear cells
PBS	Phosphate-buffered saline
PBS-T	Phosphate-buffered saline with Tween
qPCR	Quantitative real-time polymerase chain reaction
REA	Recombinant antibody
(m)RNA	(messenger) ribonucleic acid
ROS	Reactive oxygen species
RPMI	Roswell park memorial institute
Stauro	Staurosporine
Th2	T helper cell type 2
TNF	Tumor necrosis factor
TrkA	Tropomyosin receptor kinase A
TrkB	Tropomyosin receptor kinase B
TRPV1	Transient receptor potential vanilloid 1
TSLP	Thymic stromal lymphopoietin
SEM	Standard error of the mean
7-AAD	7-aminoactinomycin D
