# Supplementary material for: TRPV1 Channel in Human Eosinophils: Functional Expression and Inflammatory Modulation
Source: Int J Mol Sci. 2024 Feb 5;25(3):1922. doi: 10.3390/ijms25031922 (PMC10856050; doi:10.3390/ijms25031922)
Supplement: Supplementary file 1 [file ijms-25-01922-s001.zip › ijms-2789989-supplementary.pdf]

Supplementary method and figures

TRPV1 Channel in Human Eosinophils: Functional Expression and Inflammatory Modulation

T. Weihrauch[1], N. Gray[1,2], D. Wiebe[1], M. Schmelz[3], M.M. Limberg[1,#], U. Raap[1,4,5,#,*]

[1] Division of Experimental Allergy and Immunodermatology, Faculty of Medicine and Health Sciences, Carl von Ossietzky University Oldenburg, Oldenburg, Germany
[2] Division of Anatomy, Faculty of Medicine and Health Sciences, Carl von Ossietzky University Oldenburg, Oldenburg, Germany
[3] Department Experimental Pain Research, Medical Faculty Mannheim, University of Heidelberg, Mannheim, Germany
[4] Research Center for Neurosensory Science, Carl von Ossietzky University Oldenburg, Oldenburg, Germany
[5] University Clinic of Dermatology and Allergy, Carl von Ossietzky University Oldenburg, Oldenburg, Germany

* Correspondence: raap.ulrike@klinikum-oldenburg.de
# These authors contributed equally

Supplementary Method

Stimulation of eosinophils with IL-13 and capsaicin

Purified eosinophils were resuspended in RPMI medium (containing 10 % and 1 % PenStrep). Eosinophils were stimulated for 24 h at 37 °C and 5 % CO_2 with 0.1, 1, 10, or 100 µM capsaicin. Further, we stimulated eosinophils for 4 h at 37 °C and 5 % CO_2 with IL-13 (50 ng/ml). TRPV1 surface expression was determined using a TRPV1-PE (Biozol, Eching, Germany) antibody and flow cytometry analysis.

Figure Supplement

S1

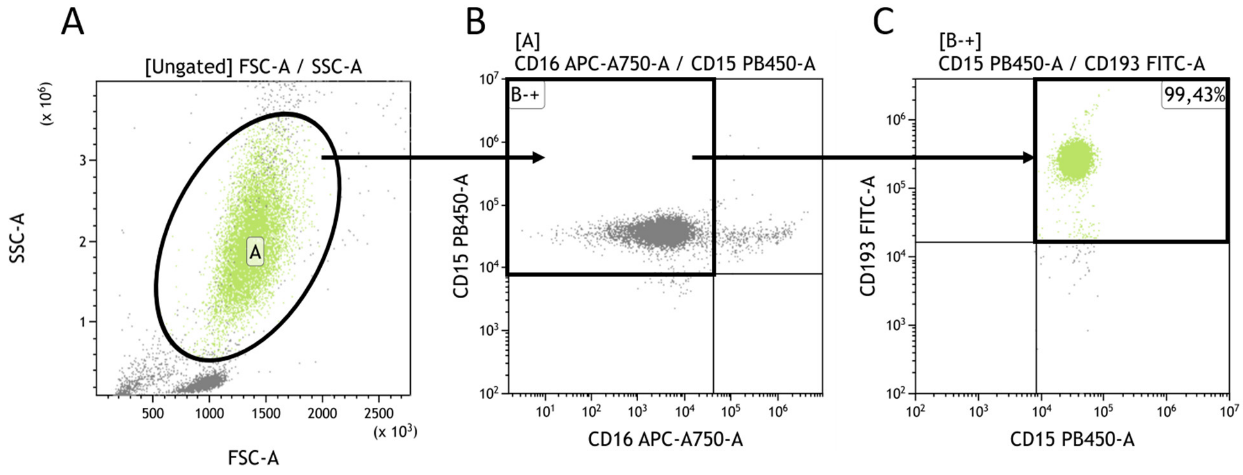

Figure S1. Gating strategy of eosinophil selection for surface content analyses. (A) Granulocyte population was selected for determining **(B)** CD15+, CD16-, and **(C)** CD193+ eosinophils. Representative dot plots of one healthy blood donor.

S2

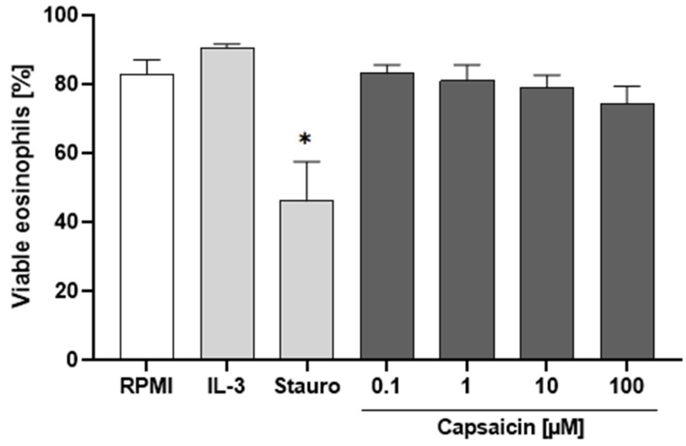

Figure S2. Detection of apoptosis 4 h after TRPV1 activation. Percentage of viable eosinophils from healthy donors after incubation with capsaicin (0.1, 1, 10, 100 µM), IL-3 (10 ng/ml) as an anti-apoptotic, staurosporine (1 µM) as a pro-apoptotic, or RPMI medium as the negative control (Co) for 4 h at 37 °C and 5 % CO_2. Eosinophils were stained with Annexin V and propidium iodide and fluorescence was measured through flow cytometry (n=5; * = $p < 0.05$; ±SEM).

S3

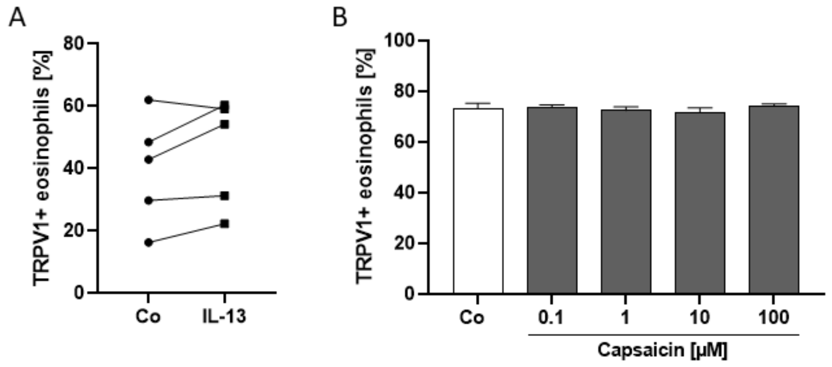

Figure S3. IL-13 and TRPV1 activation does not change TRPV1 surface content on eosinophils. (A) Percentage of TRPV1+ eosinophils of five patients after stimulation with IL-13 (50 ng/ml) or RPMI medium as the negative control (Co) for 4 h at 37 °C and 5 % CO_2. TRPV1 expression was assessed through flow cytometry (n=5). **(B)** Percentage of TRPV1+ eosinophils after stimulation with capsaicin (0.1, 1, 10, 100 µM) or RPMI medium as the negative control (Co) for 24 h at 37 °C and 5 % CO_2 (n=3; ±SEM).

S4

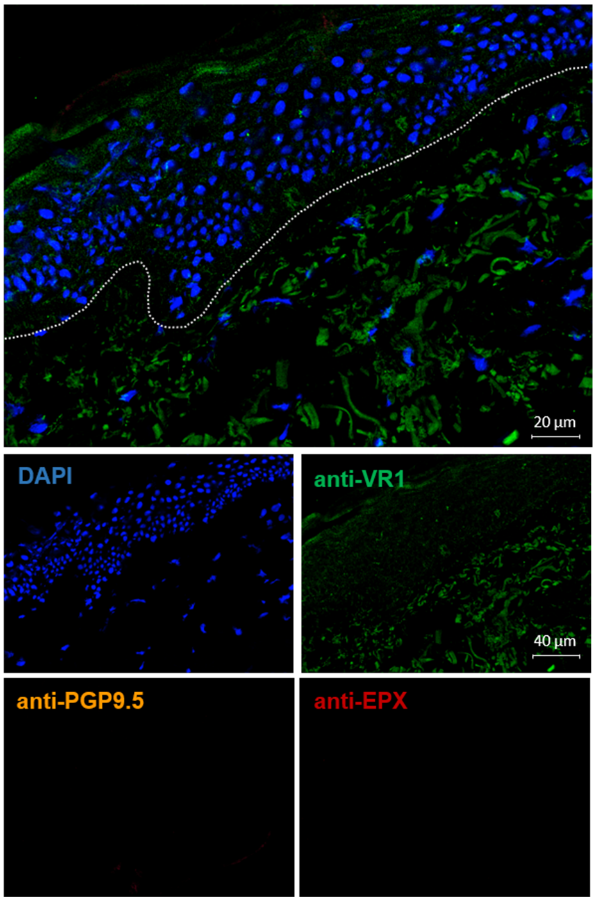

Figure S4. Immunofluorescence staining in non-inflamed skin. Skin sections obtained from healthy subjects were fixed with methanol and stained with anti-TRPV1 (green), anti-EPX (red) as the eosinophil marker, and anti-PGP9.5 (orange) as the neuronal marker. Cell nuclei were labeled with DAPI (blue). Skin section was analyzed at 40x magnification through fluorescence microscopy. Representative staining out of n=3 healthy donors.

References

1. Limberg MM, Wiebe D, Gray N, Weihrauch T, Bräuer AU et al. (2024) Functional expression of TRPV1 in human peripheral blood basophils and its regulation in atopic dermatitis. Allergy 79 (1): 225-228.
